# Supplementary material for: Smooth Interpolating Curves with Local Control and Monotone Alternating Curvature
Source: Comput Graph Forum. 2022 Oct 6;41(5):25–38. doi: 10.1111/cgf.14600 (PMC9827861; doi:10.1111/cgf.14600)
Supplement: Supplementary file 1 — Supplement Material [file CGF-41-25-s001.zip › Local-Smooth-Interpolating-MonoCurvature/extern/clothoids/submodules/Utils/docs/api-cpp/program_listing_file_Malloc.cc.html]

Program Listing for File Malloc.cc — Utils v1.2

### Navigation

- index
- toc
- Utils »
- Program Listing for File Malloc.cc

# Program Listing for File Malloc.cc¶

↰ Return to documentation for file (`Malloc.cc`)

```
/*--------------------------------------------------------------------------*\
 |                                                                          |
 |  Copyright (C) 2020                                                      |
 |                                                                          |
 |         , __                 , __                                        |
 |        /|/  \               /|/  \                                       |
 |         | __/ _   ,_         | __/ _   ,_                                |
 |         |   \|/  /  |  |   | |   \|/  /  |  |   |                        |
 |         |(__/|__/   |_/ \_/|/|(__/|__/   |_/ \_/|/                       |
 |                           /|                   /|                        |
 |                           \|                   \|                        |
 |                                                                          |
 |      Enrico Bertolazzi                                                   |
 |      Dipartimento di Ingegneria Industriale                              |
 |      Universita` degli Studi di Trento                                   |
 |      email: enrico.bertolazzi@unitn.it                                   |
 |                                                                          |
\*--------------------------------------------------------------------------*/

#ifdef __GNUC__
#pragma GCC diagnostic ignored "-Wsign-conversion"
#pragma GCC diagnostic ignored "-Wpadded"
#endif
#ifdef __clang__
#pragma clang diagnostic ignored "-Wsign-conversion"
#pragma clang diagnostic ignored "-Wweak-template-vtables"
#pragma clang diagnostic ignored "-Wc++98-compat"
#pragma clang diagnostic ignored "-Wc++98-compat-pedantic"
#pragma clang diagnostic ignored "-Wpadded"
#pragma clang diagnostic ignored "-Wdocumentation-unknown-command"
#pragma clang diagnostic ignored "-Wnon-virtual-dtor"
#pragma clang diagnostic ignored "-Wsigned-enum-bitfield"
#pragma clang diagnostic ignored "-Wpoison-system-directories"
#pragma clang diagnostic ignored "-Wexit-time-destructors"
#pragma clang diagnostic ignored "-Wglobal-constructors"
#endif

#ifndef DOXYGEN_SHOULD_SKIP_THIS

#include "Utils.hh"

#include <iostream>

namespace Utils {

  #ifndef DOXYGEN_SHOULD_SKIP_THIS
  using std::string;
  using std::mutex;
  using std::lock_guard;
  using std::exception;
  using std::exit;
  using std::cerr;
  #endif

  mutex MallocMutex;

  int64_t CountAlloc            = 0;
  int64_t CountFreed            = 0;
  int64_t AllocatedBytes        = 0;
  int64_t MaximumAllocatedBytes = 0;
  bool    MallocDebug           = false;

  string
  outBytes( size_t nb ) {
    size_t Kb = nb>>10;
    size_t Mb = Kb>>10;
    size_t Gb = Mb>>10;
    if ( Gb > 0 ) {
      size_t mb = (100*(Mb & 0x3FF))/1024;
      return fmt::format( "{}Gb (+{}Mb)", Gb, mb );
    } else if ( Mb > 0 ) {
      size_t kb = (100*(Kb & 0x3FF))/1024;
      return fmt::format( "{}Mb (+{}Kb)", Mb, kb );
    } else if ( Kb > 0 ) {
      size_t b = (100*(nb & 0x3FF))/1024;
      return fmt::format( "{}Kb (+{}bytes)", Kb, b );
    }
    return fmt::format( "{} bytes", nb );
  }

  template <typename T>
  Malloc<T>::Malloc( string const & name )
  : m_name(name)
  , m_numTotValues(0)
  , m_numTotReserved(0)
  , m_numAllocated(0)
  , m_pMalloc(nullptr)
  { }

  // - - - - - - - - - - - - - - - - - - - - - - - - - - - - - - - - - - - - - -

  template <typename T>
  void
  Malloc<T>::allocate_internal( size_t n ) {
    try {
      size_t nb;
      {
        lock_guard<mutex> lock(Utils::MallocMutex);
        nb = m_numTotReserved*sizeof(T);
        ++CountFreed; AllocatedBytes -= nb;
      }

      delete [] m_pMalloc;
      m_numTotValues   = n;
      m_numTotReserved = n + (n>>3); // 12% more values
      m_pMalloc        = new T[m_numTotReserved];

      {
        lock_guard<mutex> lock(Utils::MallocMutex);
        ++CountAlloc;
        nb = m_numTotReserved*sizeof(T);
        AllocatedBytes += nb;
        if ( MaximumAllocatedBytes < AllocatedBytes )
          MaximumAllocatedBytes = AllocatedBytes;
      }

      if ( MallocDebug )
        fmt::print( "Allocating {} for {}\n", outBytes( nb ), m_name );
    }
    catch ( exception const & exc ) {
      string reason = fmt::format(
        "Memory allocation failed: {}\nTry to allocate {} bytes for {}\n",
        exc.what(), n, m_name
      );
      printTrace( __LINE__, __FILE__, reason, cerr );
      exit(0);
    }
    catch (...) {
      string reason = fmt::format(
        "Memory allocation failed for {}: memory exausted\n", m_name
      );
      printTrace( __LINE__, __FILE__, reason, cerr );
      exit(0);
    }
    m_numTotValues = n;
    m_numAllocated = 0;
  }

  // - - - - - - - - - - - - - - - - - - - - - - - - - - - - - - - - - - - - - -

  template <typename T>
  void
  Malloc<T>::allocate( size_t n ) {
    UTILS_ASSERT(
      m_numAllocated == 0,
      "Malloc[{}]::allocate( {} ), try to allocate already allocated memory!\n",
      m_name, n
    );
    if ( n > m_numTotReserved ) allocate_internal( n );
    m_numTotValues = n;
  }

  // - - - - - - - - - - - - - - - - - - - - - - - - - - - - - - - - - - - - - -

  template <typename T>
  void
  Malloc<T>::reallocate( size_t n ) {
    if ( n > m_numTotReserved ) allocate_internal( n );
    m_numTotValues = n;
    m_numAllocated = 0;
  }

  // - - - - - - - - - - - - - - - - - - - - - - - - - - - - - - - - - - - - - -

  template <typename T>
  T *
  Malloc<T>::malloc( size_t n ) {
    UTILS_ASSERT(
      m_numAllocated == 0,
      "Malloc[{}]::malloc( {} ), try to allocate already allocated memory!\n",
      m_name, n
    );
    if ( n > m_numTotReserved ) allocate_internal( n );
    m_numTotValues = n;
    m_numAllocated = n;
    return m_pMalloc;
  }

  // - - - - - - - - - - - - - - - - - - - - - - - - - - - - - - - - - - - - - -

  template <typename T>
  T *
  Malloc<T>::realloc( size_t n ) {
    if ( n > m_numTotReserved ) allocate_internal( n );
    m_numTotValues = n;
    m_numAllocated = n;
    return m_pMalloc;
  }

  // - - - - - - - - - - - - - - - - - - - - - - - - - - - - - - - - - - - - - -

  template <typename T>
  void
  Malloc<T>::hard_free(void) {
    if ( m_pMalloc != nullptr ) {
      size_t nb;
      {
        lock_guard<mutex> lock(Utils::MallocMutex);
        nb = m_numTotReserved*sizeof(T);
        ++CountFreed; AllocatedBytes -= nb;
      }

      if ( MallocDebug )
        fmt::print( "Freeing {} for {}\n", outBytes( nb ), m_name );

      delete [] m_pMalloc; m_pMalloc = nullptr;
      m_numTotValues   = 0;
      m_numTotReserved = 0;
      m_numAllocated   = 0;
    }
  }

  // - - - - - - - - - - - - - - - - - - - - - - - - - - - - - - - - - - - - - -

  template <typename T>
  void
  Malloc<T>::memory_exausted( size_t sz ) {
    string reason = fmt::format(
      "nMalloc<{}>::operator () ({}) -- Memory EXAUSTED\n", m_name, sz
    );
    printTrace( __LINE__, __FILE__, reason, cerr );
    exit(0);
  }

  // - - - - - - - - - - - - - - - - - - - - - - - - - - - - - - - - - - - - - -

  template <typename T>
  void
  Malloc<T>::must_be_empty( char const * const where ) const {
    if ( m_numAllocated < m_numTotValues ) {
      string tmp = fmt::format(
        "in {} {}: not fully used!\nUnused: {} values\n",
        m_name, where, m_numTotValues - m_numAllocated
      );
      printTrace( __LINE__,__FILE__, tmp, cerr );
    }
    if ( m_numAllocated > m_numTotValues ) {
      string tmp = fmt::format(
        "in {} {}: too much used!\nMore used: {} values\n",
        m_name, where, m_numAllocated - m_numTotValues
      );
      printTrace( __LINE__,__FILE__, tmp, cerr );
    }
  }

  template class Malloc<char>;
  template class Malloc<uint16_t>;
  template class Malloc<int16_t>;
  template class Malloc<uint32_t>;
  template class Malloc<int32_t>;
  template class Malloc<uint64_t>;
  template class Malloc<int64_t>;
  template class Malloc<float>;
  template class Malloc<double>;

  template class Malloc<void*>;
  template class Malloc<char*>;
  template class Malloc<uint16_t*>;
  template class Malloc<int16_t*>;
  template class Malloc<uint32_t*>;
  template class Malloc<int32_t*>;
  template class Malloc<uint64_t*>;
  template class Malloc<int64_t*>;
  template class Malloc<float*>;
  template class Malloc<double*>;

} // end namespace lapack_wrapper

#endif
```

### Quick search

### Table of Contents

- rang - Colors for your Terminal
- Zstr
- C++ API

«
hide menu

menu
sidebar
»

### Navigation

- index
- toc
- Utils »
- Program Listing for File Malloc.cc

© Copyright 2021, Enrico Bertolazzi.
Created using Sphinx 4.2.0.
